# A Silk Fibroin Nanoparticle Hydrogel Loaded With NK1R Antagonist Has Synergistic Anti‐Inflammatory and Reparative Effects on Dry Eye Disease

**DOI:** 10.1002/advs.202404835

**Published:** 2025-02-22

**Authors:** Bo Gong, Yi Liu, Huan Li, Xueming Ju, Dongfeng Li, Yuhao Zou, Xiaoxin Guo, Kai Dong, Jialing Xiao, Weijia Wu, Renjie Chai, Ruifan Zhang, Man Yu

**Affiliations:** ^1^ Department of Ophthalmology Sichuan Academy of Medical Sciences & Sichuan Provincial People's Hospital University of Electronic Science and Technology of China Chengdu 610000 China; ^2^ Human Disease Genes Key Laboratory of Sichuan Province and Institute of Laboratory Medicine Sichuan Academy of Medical Sciences & Sichuan Provincial People's Hospital University of Electronic Science and Technology of China Chengdu 610000 China; ^3^ Research Unit for Blindness Prevention of Chinese Academy of Medical Sciences (2019RU026) Sichuan Academy of Medical Sciences & Sichuan Provincial People's Hospital University of Electronic Science and Technology of China Chengdu 610000 China; ^4^ Department of Ophthalmology Deyang People's Hospital Deyang 618000 China; ^5^ School of Medicine University of Electronic Science and Technology of China Chengdu 610000 China; ^6^ State Key Laboratory of Digital Medical Engineering, Department of Otolaryngology‐Head and Neck Surgery Zhongda Hospital School of Life Sciences and Technology School of Medicine, Advanced Institute for Life and Health Jiangsu Province High‐Tech Key Laboratory for Bio‐Medical Research Southeast University Nanjing 210096 China; ^7^ Co‐Innovation Center of Neuroregeneration Nantong University Nantong 226001 China; ^8^ Department of Neurology Aerospace Center Hospital School of Life Science Beijing Institute of Technology Beijing 100081 China; ^9^ Department of Otolaryngology Head and Neck Surgery, Sichuan Provincial People's Hospital School of Medicine University of Electronic Science and Technology of China Chengdu 610072 China; ^10^ Southeast University Shenzhen Research Institute Shenzhen 518063 China

**Keywords:** anti‐inflammatory, dry eye disease, NK1R antagonist, repair, silk fibroin nanoparticles

## Abstract

Dry eye disease (DED) is a multifactorial illness affecting tears and the ocular surface. The neurokinin 1 receptor (NK1R) is a target for controlling T helper 17 (Th17) and regulatory T cell (Treg) imbalances. This work creates a silk fibroin (SF) nanoparticle hydrogel that targets NK1R with CP‐99,994 (CP). Combining CP and SF to generate stable nanoparticles while integrating a flexible hydrogel material results in a sustained‐release ophthalmic drop formulation (SF@CP@Gel), which provides a long‐lasting ocular formulation with anti‐inflammatory and reparative properties. SF@CP@Gel could maintain a stable CP concentration for 25 h with detectable biological activity. The cell counting kit‐8 and 2,7‐DHL‐DA results reveal that SF@CP@Gel has no cytotoxic effect on human corneal epithelial cells (HCECs) and decreases the reactive oxygen species level in oxidatively damaged HCECs. Cell scratch assays demonstrate that SF@CP@Gel can greatly increase HCEC migration and proliferation within 24 h. Furthermore, in vivo therapy with topical SF@CP@Gel twice daily markedly reduce clinical symptoms by reducing the amount of pathogenic Th17 cells while efficiently restoring Treg activity. In summary, this work reveals that SF@CP@Gel might attenuate DED by inhibiting NK1R‐mediated SP signaling and thereby modulating the Th17/Treg ratio, a potential anti‐inflammatory and repair treatment method for DED.

## Graphical abstract

This study develops a silk fibroin (SF) nanoparticles hydrogel targeting neurokinin 1 receptor with CP‐99994 (CP). Combining CP and SF to form stable nanoparticles while incorporating a flexible hydrogel material results in a sustained‐release ophthalmic drop formulation (SF@CP@Gel), which provides a long‐lasting ophthalmic formulation with anti‐inflammatory and reparative activities. In brief, SF@CP@Gel is a promising anti‐inflammatory and repair therapeutic strategy for dry eye disease.

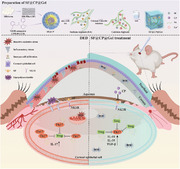

## 1 Introduction

Dry eye disease (DED) is a common and chronic ocular surface disease that affects 5%–50% of the population worldwide.^[^ ^1^ ^]^ Its main features are chronic inflammatory infiltration of the conjunctiva, cornea, and tear film with a range of tissue damage, including loss of epithelial and goblet cells, early tear film breakup, disruption of the corneal epithelial barrier, and neurosensory abnormalities.^[^ ^2^, ^3^ ^]^ Although the clinical features of DED are well described, its pathogenesis remains unclear. Dysfunctional T‐cell‐dependent immunity significantly drives the inflammatory process of DED. Studies have confirmed the central role of homeostasis between interleukin 17 (IL‐17)‐secreting T helper 17 (Th17) cells and regulatory T cells (Tregs) in tissue inflammatory damage.^[^ ^4^, ^5^, ^6^, ^7^ ^]^ However, few studies have emphasized the Th17/Treg balance in DED patients.

Substance P (SP) is an 11‐amino acid peptide member of the tachykinin family secreted by healthy corneal nerves at the ocular surface.^[^ ^8^ ^]^ SP reportedly acts as a double‐edged sword in the cornea, and its physiological levels promote corneal wound healing through overexpression of SP;^[^ ^9^, ^10^, ^11^, ^12^ ^]^ however, this effect might be reversed and mediate neurogenic inflammation by inducing Th17 generation.^[^ ^13^, ^14^ ^]^ In addition, high levels of SP can lead to Treg dysfunction.^[^ ^15^ ^]^ This process requires the interaction of SP with its receptor, and the neurokinin 1 receptor (NK1R) has the highest affinity.^[^ ^16^ ^]^ These findings suggest that the SP/NK1R pathway may be a key target for regulating Th17/Treg homeostasis. Blocking the SP/NK1R pathway with CP‐99994 (CP) and L‐733060 can inhibit the maturation of antigen‐presenting cells (APCs) in the cornea and draining lymph nodes (DLNs), thereby preventing the activation of Th17 cells and the infiltration of inflammatory factors.^[^ ^17^ ^]^ Moreover, the use of the NK1R antagonist Spantide I in DED mice effectively preserved Treg function while suppressing the Th17 response.^[^ ^15^ ^]^ Numerous in vivo experiments have shown that NK1R antagonists can regulate immune cell activation and inhibit corneal neovascularization and pathological lymphangiogenesis in vivo.^[^ ^15^, ^17^, ^18^, ^19^ ^]^ Overall, the SP/NK1R pathway may be an effective therapeutic target for alleviating the severity of DED and has tremendous potential for transformation and application.

However, due to drug delivery barriers, traditional ocular drug delivery methods (such as eye drops) exhibit low bioavailability and unavoidable toxic side effects.^[^ ^20^, ^21^ ^]^ Thus, there is an urgent need to develop sustained‐release, low‐toxicity, and high‐permeability therapeutic strategies to target the SP/NK1R pathway. In response to these demands, a variety of ocular drug delivery systems, such as silk fibroin (SF), have been developed in recent years. SF is a natural protein polymer with excellent biocompatibility, stability, and degradability, which can be processed by different methods to produce various types of microcarrier drug delivery systems, including films, hydrogels, foams, scaffolds, and microparticles.^[^ ^22^, ^23^ ^]^ Among them, SF hydrogels have been shown to have excellent biocompatibility and structural stability, with the ability to achieve sustained drug release.^[^ ^24^ ^]^ In addition, nanoparticles have become novel ocular drug carriers for improving ocular bioavailability due to their small particle size, which can increase the corneal residence time and reduce adverse drug reactions.^[^ ^25^, ^26^, ^27^ ^]^ Previous studies, such as the work by Feng et al.,^[^ ^28^ ^]^ demonstrated that combining SF microspheres with alginate hydrogels could effectively prolong the release of growth factors and achieve significant effects in myocardial repair. Building on this foundation, our study further explored the potential of SF in ocular drug delivery, leveraging its excellent biocompatibility, anti‐inflammatory properties, and sustained release capabilities to develop a long‐acting hydrogel system for the treatment of DED.

In this study, we developed a new targeted drug against the SP/NK1R pathway by utilizing an SF‐loaded NK1R antagonist (CP99, 994) to construct a drug‐loaded system of nanoparticles while incorporating a hydrogel for ocular drug delivery. Our results suggest that SF@CP@Gel is a safe and effective anti‐inflammatory repair drug for DED that can regulate the Th17/Treg balance, repair damaged tissue of the keratoconjunctiva, and significantly alleviate experimental DED symptoms and signs. We acknowledge that numerous studies have investigated the use of nanoparticle‐loaded hydrogels for ocular surface drug delivery.^[^ ^29^ ^]^ However, our study uniquely combines SF nanoparticles with an NK1R pathway antagonist to develop a long‐acting drug delivery system capable of regulating the Th17/Treg balance and promoting ocular surface tissue repair, providing a novel solution for the treatment of DED.^[^ ^29^ ^]^

## 2 Results

### 2.1 Preparation and Characterization of Drug‐Loaded SF Gels

**Figure 1:**
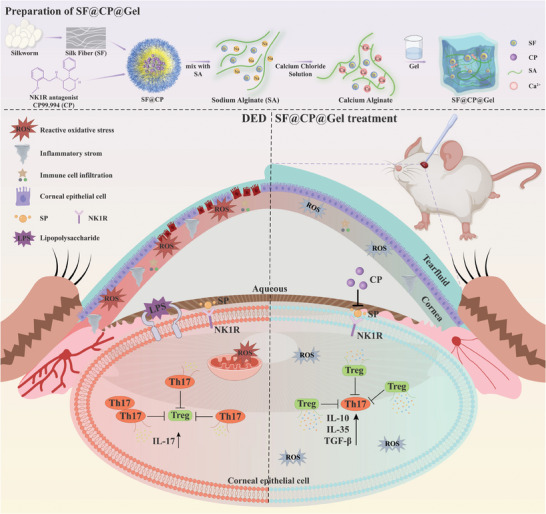
Schematic illustration of the preparation of SF@CP@Gel and its anti‐inflammatory and antioxidant effects on the ocular surface via the SP/NK1R pathway, which stabilizes the tear film and promotes corneal epithelial repair in mice with DED.

**Figure 2:**
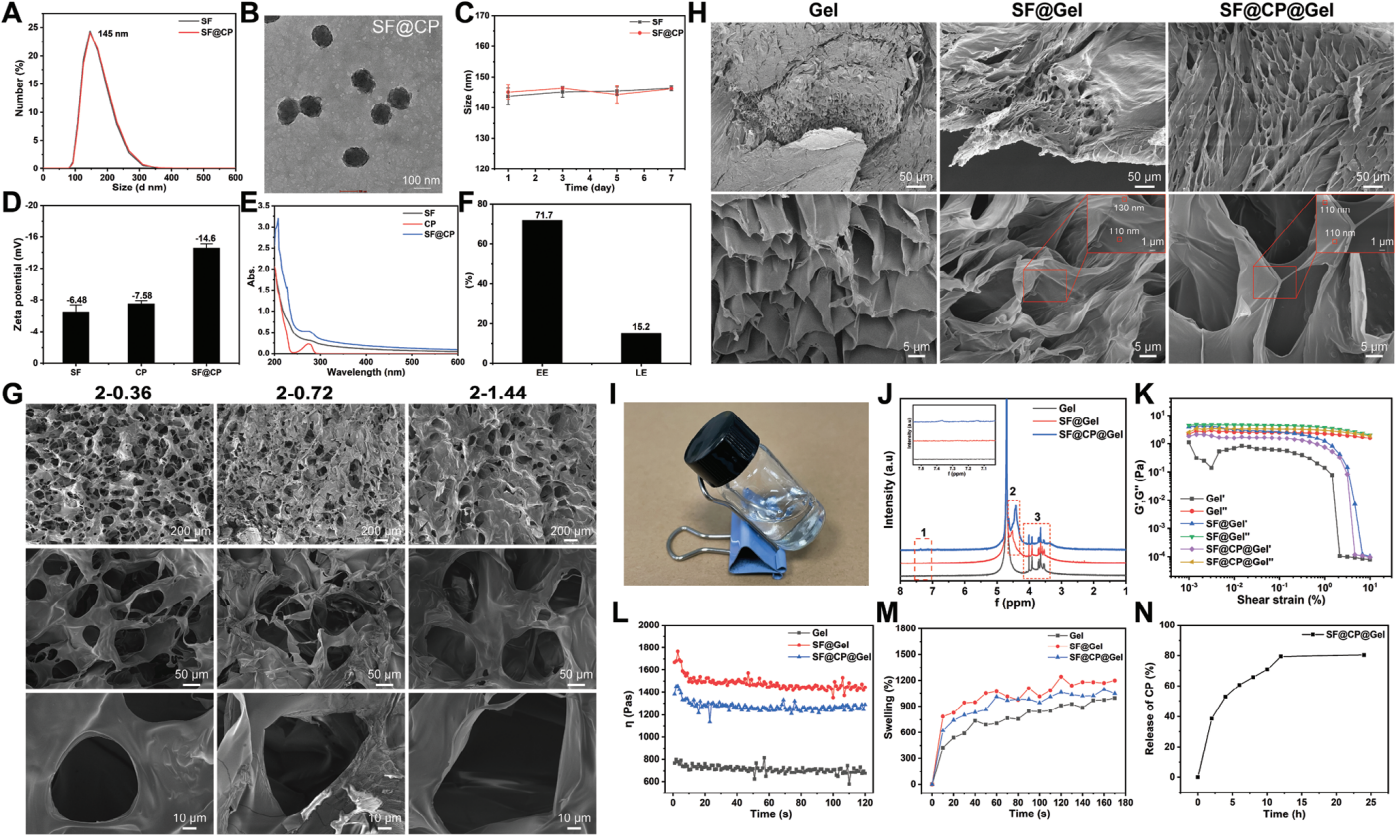
Characterization of drug‐carrying nanosystems and physicochemical properties of drug‐carrying SF hydrogels. A) Hydrodynamic diameters of SF@CP as measured by DLS. B) TEM images of SF@CP; scale bar = 100 nm. C) Size stability of SF@CP within 7 days. D) Zeta potentials of SF, CP, and SF@CP. E) UV spectrum analysis of SF, CP, and SF@CP. F) Drug‐loading efficiency and encapsulation efficiency of CP from SF@CP. G) Scanning electron microscope (SEM) images of gels; scale bars = 200, 50, and 10 µm. H) SEM images of gels; scale bars = 50 and 5 µm. I) Image of SF@CP@Gel. J) Proton NMR spectra of Gel, SF@Gel, and SF@CP@Gel. K) A 15‐mL sample of Gel, SF@Gel, and SF@CP@Gel was used to characterize the rheological properties of the hydrogel using a micro infrared rheometer, and oscillatory rate sweep tests were performed at 25 °C ± 0.2 °C with γ = 0%–1000% and f = 1 Hz. L) Temperature (25 °C), strain (γ = 1%), and frequency (1 Hz) were fixed to obtain viscosity‐time relationship curves of Gel, SF@Gel, and SF@CP@Gel. M) Swelling ratio of Gel, SF@Gel, and SF@CP@Gel. N) UV spectrophotometry to determine drug release from this drug delivery system. The absorbance at 275 nm in the supernatant was determined by UV spectroscopy by calculating the release at different times based on the standard curve. The cumulative method was used to calculate the release at different times, hence the release rate of CP from SF@CP@Gel.

The experimental flowchart is shown in **Figure** **1**. We first characterized the drug‐carrying nanosystems to determine their stable drug‐carrying and drug‐releasing properties. Dynamic light scattering (DLS) measurements of the hydrodynamic diameter of SF@CP particles revealed that the particles were 122 nm in diameter (**Figure** **2**A). According to the transmission electron microscopy (TEM) image, SF@CP was spherical with a size of ≈120 nm, which was consistent with the particle size measurements (Figure 2B). The stability of drug‐carrying nanoparticles directly affects drug release performance. As shown in Figure 2C, the stability test results of SF@CP showed that its size remained stable at 145 nm for 7 days, demonstrating its ability to circulate and sustain release in vivo for a long period. To determine whether the CP drug was successfully loaded, we performed potentiometric tests and ultraviolet (UV) spectral analysis on SF, CP, and SF@CP. As shown in Figure 2D, the absolute value of the potential of SF@CP was significantly greater than that of SF and CP solutions alone, indicating that the drug‐loaded nanoparticle system was more stable. The UV spectra showed that the characteristic absorption peak of the CP drug appeared at 275 nm in the SF@CP group compared to the SF alone group (Figure 2E). The drug loading and encapsulation rates of CP were 71.7% and 15.2%, respectively (Figure 2F). These results indicated that the CP drug was successfully encapsulated in the SF nanoparticles.

We further characterized the physicochemical properties of the drug‐loaded SF hydrogel to determine its ability to stabilize drug loading and control drug release. As shown in Figure 2G,H, the SF@CP@Gel group exhibited a 3D network structure with mutual cross‐linking and ≈120 nm particles, which was consistent with the particle size results of the drug‐loaded SF nanoparticles, proving that the SF hydrogel was successfully loaded with the CP drug. Figure 2I shows a physical image of the drug‐loaded SF gel, which showed a transparent and homogeneous gel state without obvious particle precipitation, indicating that the drug‐loaded nanoparticles were uniformly dispersed in the gel material and had good fluidity. The structural analyses of Gel, SF@Gel, and SF@CP@Gel are shown in Figure 2J. The signal peaks between 5.5 and 3.0 ppm were attributed to the plasmonic signals on the alginate (SA) backbone, whereas those between 5.5 and 3.0 ppm were attributed to the incorporation of SF, which decreased the amount of SA. The proton peak at 4.5 ppm was attributed to the proton peak of leucine in the SF. The signal of SF@CP@Gel between 5.5 and 3.0 ppm was further weakened due to the decrease in alginate content by the addition of SF. A new proton peak appeared at 7.37 ppm, which was the peak of the benzene ring in CP, and the peak of leucine from 4.5 to 4.3 ppm, which was due to the formation of a π─π bond between the benzene ring in the drug and the carboxyl group in the leucine. The electron cloud was biased toward the carboxyl group, which increased the density of the electron cloud around the leucine of the SF and decreased the chemical shifts due to the enhancement of the shielding effect. The rheological properties of Gel, SF@Gel, SF@CP, and SF@CP@Gel are shown in Figure 2K. With increasing shear stress, the energy storage modulus (G′) and loss modulus (G″) were comparable, as both were distributed between 0.5 and 10 Pa, indicating that the samples possessed both solid and liquid properties. In addition, G′ < G″ indicated that the sample was mainly a liquid with strong fluidity. With increasing shear strain (γ), G′ decreased rapidly when γ was >1%, and then the curve tended to stabilize, which indicates that there was a colloid initially and then a local or overall collapse of the sample, which completely transformed into a liquid state.

In summary, it can be proven that the gel is a semisolid material, but the liquid property is dominant. Moreover, the energy storage modulus G′ of the liquid gel is always smaller than the loss modulus G″, and there is no intersection point, which means that the gel does not have a symbolic “gel point.” This is mainly because the hydrogel is mostly liquid, and with increasing shear strain, the gel structure is further damaged, the energy storage modulus G′ decreases, and the structural stability weakens, resulting in no intersection point. Subsequently, the frequency and shear strain were fixed at room temperature to observe the change in viscosity over time, and the results are shown in Figure 2L. The viscosity of the gels was stable within 120 s. The viscosity of SF and drug‐loaded SF increased significantly after the addition of SF because many hydroxyl and amine groups on the SF could combine with the carboxyl and hydroxyl groups on the side chain of sodium alginate through hydrogen bonding. The viscosity of the drug‐loaded SF nanoparticle gels decreased slightly compared to that of the SF gels, probably because parts of the SFs were bound to the drug, thus reducing the binding to sodium alginate and leading to a decrease in viscosity, which was consistent with the nuclear magnetic resonance (NMR) spectroscopy results. The dissolution property assay is important for predicting drug release and adhesion ability. Our results are shown in Figure 2M. The gel rapidly absorbed water and swelled in phosphate‐buffered saline (PBS), and the swelling rates were 400%, 600%, and 800% at 10 s, respectively. The swelling rate of SF gel was the largest, which might be caused by the formation of a micelle‐like structure by the many hydrophobic and hydrophilic groups on the SF side chains in addition to the mutual electrostatic repulsive effect of the ionized coo‐groups of the sodium alginate side chains. The release of the CP in the drug‐loaded SF gel was then tested using UV, and the test curve is shown in Figure 2N. The test results showed that 60% of the CP drug could be released at 6 h; at 12 h, 60% of the CP drug could reach release equilibrium, and its cumulative release reached 80%. The CP drug could maintain its concentration for 25 h.

### 2.2 In Vitro Cytotoxicity of the Drug‐Loaded SF Hydrogels

**Figure 3:**
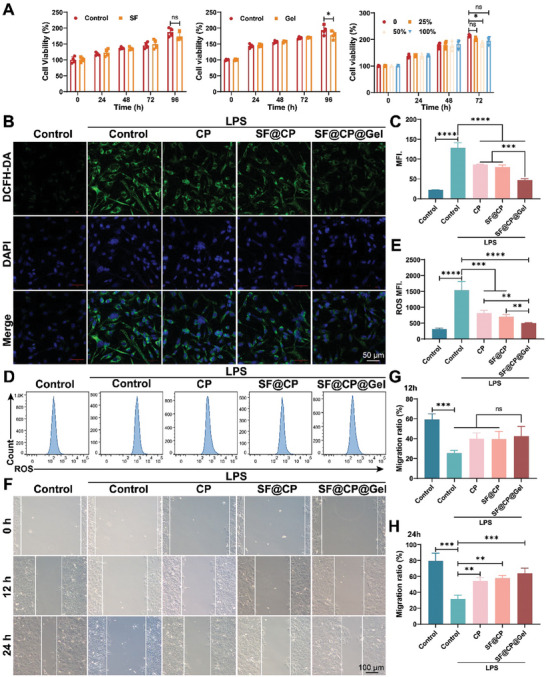
In vitro cytotoxicity and antioxidant and cell migration tests of different drug‐carrying biomaterials. SF NPs, SF@CP@Gel hydrogels, and different concentrations of SF@CP@Gel extracts prepared in advance were added to HCECs and incubated for 24 h. Subsequently, CCK‐8 reagent was added, and the cells were incubated for 0, 24, 48, 72, or 96 h. A) CCK‐8 assay results and statistical analysis of HCECs after coculture with SF NPs, SF@CP@Gel hydrogels, or different concentrations of gel extracts. HCECs were treated with 1 mg mL^−1^ lipopolysaccharide (LPS) for 24 h to induce oxidative stress and then treated with CP, SF@CP, or the SF@CP@Gel hydrogel for 24 h. HCECs were then incubated with 20 µM 2,7‐dichlorodihydrofluorescein diacetate (DCFH‐DA) solution for green fluorescence imaging. Untreated cells were used as controls. B) The generation of ROS was monitored using a fluorescence microscope (green). Scale bar = 50 pm. C) Semiquantitative analysis of ROS generation monitored using DCFH‐DA. The results are expressed as the mean fluorescence intensity (MFI) of DCF. D) The generation of ROS was monitored using a flow cytometer. E) Semiquantitative analysis of ROS generation monitored using a flow cytometer. F) A sterile 200 µl plastic pipette tip was used to make scratches across the HCEC surface to create artificial scratches. CP, SF@CP, and SF@CP@Gel hydrogels were added for coincubation, and images of the same scratch area were taken at 0, 6, 18, and 24 h to determine scratch closure. The scratch area was measured using ImageJ after the cells were cocultured with different groups at G) 12 h and H) 24 h. (n = 4. One‐way ANOVA and Two‐way ANOVA were used for the statistical analysis: ns, no significance, *p < 0.05, **p < 0.01, ***p < 0.001, ****p < 0.0001; Data are presented as means ± SEM).

A major prerequisite for novel drug development is harmlessness or low cytotoxicity. We evaluated the biocompatibility of the drug‐carrying materials and tested the viability of human corneal epithelial cells (HCECs) using the cell counting kit‐8 (CCK‐8) assay. As shown in **Figure** **3**A, the optical density (OD) values of the blank and SF groups were nearly the same, indicating that the SF materials did not affect cell viability. The effect of the gel material on cell viability was then further investigated. Figure 3A shows that over time, the cells in the blank and gel groups steadily proliferated without any significant difference, and the cells steadily proliferated with increasing time at different SF@CP@Gel extract concentrations without any significant decrease compared to those in the blank group, thus indicating that both the nano and gel materials have excellent safety.

### 2.3 Effective Antioxidant and Pro‐Cell Migration Capacity

We tested the antioxidant and migratory capacities of different subgroups of drugs using well‐grown isolated primary HCECs. First, the degree of cellular oxidative damage was assessed by detecting the content of reactive oxygen species (ROS), as shown in Figure 3B. Combining the dichlorofluorescein (DCF) probe with ROS can result in green fluorescence under a confocal microscope; the greater the intensity of green fluorescence is, the more reactive the oxygen species becomes, and the nucleus of the cell is labeled with 4′,6‐diamidino‐2‐phenylindole (DAPI). As seen from the cell confocal images, the ROS fluorescence levels of the three treatment groups (CP treatment group, SF@CP treatment group, and SF@CP@Gel treatment group with CP concentration of 5 µg mL^−1^) significantly decreased compared with those of the control group, and Figure 3C shows their corresponding statistical results. The ROS content in the SF@CP@Gel group was significantly reduced compared with that in the other two groups (*p* < 0.001). Flow cytometry was further utilized to detect the green fluorescence of the probes through the fluorescein isothiocyanate (FITC) channel to quantitatively determine the degree of oxidative damage in the cells (Figure 3D), and the corresponding semiquantitative results are shown in Figure 3E. The results showed that the ROS levels of all three treatment groups significantly decreased compared with those of the control group, with no significant difference between the treatment groups but still demonstrated a decreasing trend. To further determine the effect of the drug‐loaded SF gels on cell migration ability, we performed cell scratch experiments, and the results are shown in Figure 3F. The corresponding quantitative results at 12 and 24 h are shown in Figure 3G,H, respectively. At 12 h, the cell migration rate tended to increase in each treatment group; however, the difference was not statistically significant. At 24 h, the cell migration rates of all three treatment groups were significantly greater than that of the control group, with no significant difference between the groups.

### 2.4 In Vivo Assessment of Treatment Efficacy

**Figure 4:**
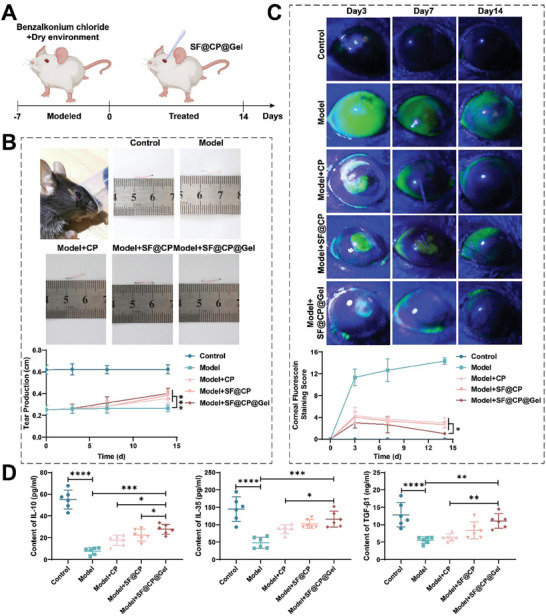
Administration timeline and in vivo therapeutic effects of different drug delivery systems. Mice that did not receive any treatment composed the normal control group, and those in which the dry eye model was successfully established only received 10 µL of PBS treatment composed the model group. The mice in the treatment group included those in the CP treatment group, SF@CP treatment group, and SF@CP@Gel treatment group. Topical administration to both eyes was administered once a day in the morning or evening at a dose of 10 µL for 14 days. A) Experimental timeline. B) Tear secretion and statistical analysis of the control group, DED model group, CP treatment group, SF@CP treatment group, and SF@CP@Gel treatment group. C) CFS and statistical analysis of different groups: the control group, DED model group, CP treatment group, SF@CP treatment group, and SF@CP@Gel treatment group. D) IL‐10, IL‐35, and TGF‐β1 levels in conjunctival tissues were measured using ELISA. (n = 14 mice per groups. One‐way ANOVA was used for the statistical analysis: ns, no significance, *p < 0.05, **p < 0.01, ***p < 0.001, ****p < 0.0001; Data are presented as means ± SEM).

The timeline of drug administration to the experimental mice is shown in **Figure** **4**A. The therapeutic efficacy of the experimental dry eye mouse model was evaluated by determining the clinical indicators, such as tear secretion and corneal fluorescein staining (CFS), after treatment in the CP group and the SF@CP and SF@CP@Gel groups. As shown in Figure 4B, tear secretion in the normal and model groups was stable, and the lengths of the cotton threads were 0.62 and 0.25 mm, respectively, indicating that the dry eye mouse model was successfully constructed. The length of the cotton threads in the three treatment groups gradually increased with time, and after 14 days of treatment, the lengths of the cotton threads in the SF@CP and SF@CP@Gel treatment groups were similar, and both were close to 0.39 mm. The extent of corneal epithelial damage was assessed using the CFS method, and the corneal epithelium was stained with fluorescein and then visualized and photographed under the cobalt‐blue light of a slit lamp (Figure 4C). The corneal epithelial defects manifested as a yellowish‐green coloring area. The scoring method was based on the National Eye Institute (NEI) criteria, which have the best reliability and reproducibility.^[^ ^30^ ^]^ The CFS scores are shown in Figure 4C, where a significant decrease in scores was observed in all three treatment groups on the third day of drug administration. After 14 days of treatment, the CFS of the SF@CP@Gel group decreased by 93.2% compared with that of the DED group, and the therapeutic effect of SF@CP@Gel was significantly greater than that of free CP (CFS decreased by 79.07%) and SF‐loaded CP (CFS decreased by 81.39%).

The function of Treg cells was examined using enzyme‐linked immunosorbent assay (ELISA), which revealed changes in the inhibitory cytokines IL‐10, IL‐35, and TGF‐β1 before and after treatment. As shown in Figure 4D, there were statistically significant differences in the IL‐10, IL‐35, and TGF‐β1 levels between the DED model group and the normal group (*p* < 0.0001), indicating that the dry eye model was successfully constructed. The IL‐10 level in the SF@CP@Gel treatment group was 5.01 times greater than that in the dry eye model without drug treatment group (*p* < 0.001), and there were significant differences between the SF@CP treatment group and the SF@CP@Gel treatment group (*p* < 0.05). The IL‐35 level was significantly upregulated by treatment in the three different treatment groups. After SF@CP@gel (*p* < 0.0001) treatment, the level of TGF‐β1 in DED mice showed a statistically significant upward trend, although there was no significant difference between the other two treatment groups and DED mice. In addition, in order to evaluate the biological safety of drugs in vivo, the histological changes of the heart, liver, spleen, lungs, and kidneys in the control group, DED model group, CP treatment group, SF@CP treatment group, and SF@CP@Gel treatment group were analyzed by hematoxylin‐eosin staining (see Figure S3, Supporting Information for details).

### 2.5 Histological Assessment of Treatment Effectiveness

**Figure 5:**
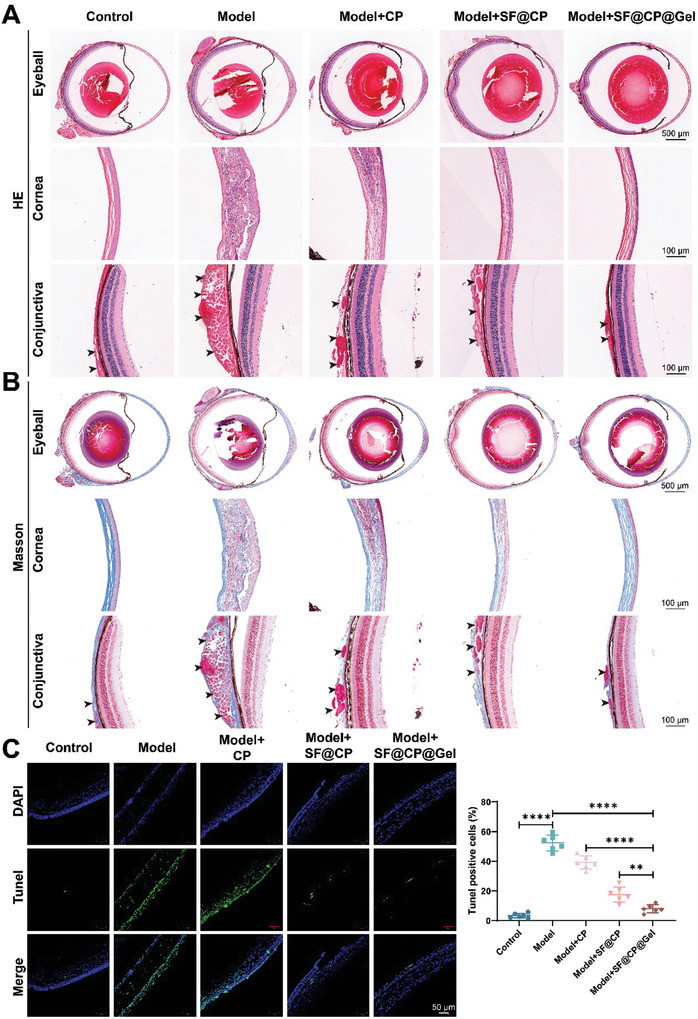
Histological analysis of different drug delivery systems. A) The HE staining of eyeballs, corneas, and conjunctivae from the control group, DED model group, CP treatment group, SF@CP treatment group, and SF@CP@Gel treatment group. B) Masson staining of eyeballs, corneas, and conjunctivae from the control group, DED model group, CP treatment group, SF@CP treatment group, and SF@CP@Gel treatment group. C) Fluorogram of TUNEL staining and calculation of TUNEL staining positivity in different groups. Moreover, through optimal cutting temperature (OCT) embedding and frozen sectioning, dihydroethidium probes were used to detect ROS levels in the cornea. The results showed that compared to the control group, the model group exhibited a significant increase in red fluorescence signals and a notable rise in ROS‐positive cells, indicating a significant elevation in corneal ROS levels. Compared to the model group, the CP, SF@CP, and SF@CP@Gel groups showed reduced red fluorescence signals and a decrease in ROS‐positive cells, leading to a significant reduction in corneal ROS levels, with statistical differences observed (n = 14 mice per groups. One‐way ANOVA was used for the statistical analysis: **p < 0.01, ****p < 0.0001; Data are presented as means ± SEM).

Eyeball, cornea, and conjunctiva specimens were collected for histological examination to determine the recovery of damaged tissues in mice with experimental dry eye after drug treatment. The HE staining results are shown in **Figure** **5**A. Compared with those in the normal group, the corneal epithelial cells of the mice in the dry eye model group were disorganized, with a marked increase in the number of cells and abnormal morphology; the collagen fibers of the corneal stroma layer were disorganized, swollen, and intertwined with the epithelium; and the conjunctiva was severely damaged, with poorly aligned cells accompanied by defects and increased spacing of cells. The corneal epithelial cells were poorly arranged and were accompanied by defects, increased cell spacing, a loose structure, and severe damage. After treatment in the different groups, the number of epithelial cells in the cornea gradually decreased; the collagen fibers in the stroma and epithelial layers were clearly structured and neatly arranged, and swelling disappeared. The conjunctival cells gradually became neatly arranged and dense, and the spacing between the cells decreased. The results of Masson staining are shown in Figure 5B, in which the collagen fibers in the stroma layer of the normal group were neatly arranged, the epithelial layer in the model group was strongly intertwined with the stroma layer and the corneal collagen fibers, and there was swelling. In the normal group, the collagen fibers of the stratum corneum were poorly aligned, while in the model group, the collagen fibers of the epithelium and stratum corneum were disorganized and swollen. After the different treatments, the collagen fibers in the stromal and epithelial layers became structurally distinct and then gradually aligned. The swelling disappeared, and the conjunctival cells gradually aligned and became densely packed, with a decrease in cell spacing. According to the above tissue staining images, the corneal morphology of the SF@CP@Gel treatment group was similar to that of the normal group, indicating that the treatment effect of the SF@CP@Gel treatment group was better than that of the CP treatment group and the SF@CP treatment group and was able to restore the damaged tissues effectively. Terminal deoxynucleotidyl transferase‐mediated 2′‐deoxyuridine 5′‐triphosphate (dUTP) nick end labeling (TUNEL) experiments were performed to further assess tissue apoptosis before and after treatment. Figure 5C shows the fluorescence graph of TUNEL staining and the calculation results of the percentage of TUNEL‐positive cells. The model group showed many apoptotic corneal epithelial cells with strong green fluorescence, and the percentage of apoptotic cells was significantly different from that of the normal group (*p* < 0.0001), indicating that the dry eye model was successful. There were significant differences between the model group and the other three treatment groups (*p* < 0.05, 0.0001, and 0.0001, respectively). After free CP, SF@CP, and SF@CP@Gel treatment, the percentage of positive cells decreased by 26.29% (*p* < 0.05), 63.98% (*p* < 0.0001), and 87.43%, respectively. This indicates that CP, SF@CP, and SF@CP@Gel possess antioxidant effects that can alleviate oxidative damage to the cornea caused by dry eye, with SF@CP@Gel showing the most significant effect (Figure S4, Supporting Information).

### 2.6 SF@CP@Gel Regulates the SP/NK1R Signaling Pathway

**Figure 6:**
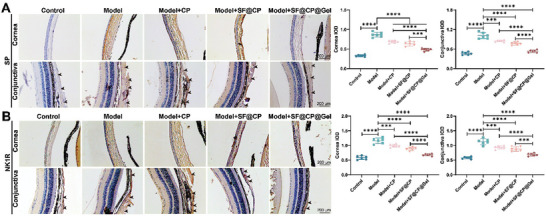
Immunohistochemical analysis of SP and NK1R expression in the cornea and conjunctiva. Anti‐NK1R antibody or anti‐SP antibody was added to the cornea and conjunctiva tissue, and DAPI restaining was performed. The sections were observed and photographed A) under a fluorescence microscope and quantitatively analyzed to obtain the IOD using ImageJ software B) (n = 14 mice per groups. One‐way ANOVA was used for the statistical analysis: ***p < 0.001, ****p < 0.0001; Data are presented as means ± SEM).

To further confirm the efficacy of SF@CP@Gel, we stained SP and NK1R in the cornea and conjunctiva before and after treatment with a DAPI complex staining agent (blue is negative, and yellow is positive) and observed them using fluorescence microscopy (**Figure** **6**). The positive results were quantified using ImageJ to obtain the integrated ODs (IODs) of SP (Figure 6A) and NK1R (Figure 6B). In both the cornea and conjunctiva, the IODs of SP and NK1R were significantly different between the normal control and model groups (all *p* < 0.0001), again demonstrating the success of the model in our dry eye model mice. As shown in Figure 6, the downregulation of both SP and NK1R in the cornea and conjunctiva was statistically significant between the dry eye model group and each treatment group. The IOD of SPs in the corneas of DED mice decreased by 43.74% (*p* < 0.0001) after SF@CP@Gel treatment, 18.40% (*p* < 0.001) after CP treatment, and 25.26% (*p* < 0.01) after SF@CP treatment. In the conjunctiva DED model, the IOD of SP decreased by 48.30% (*p* < 0.0001) after SF@CP@Gel treatment, 16.36% (*p* < 0.001) after CP treatment, and 23.40% (*p* < 0.01) after SF@CP treatment. The level of NK1R in the corneas of DED mice decreased by 46.12% (*p* < 0.0001) in the SF@CP@Gel group, 17.77% (*p* < 0.0001) after CP treatment, and 25.89% (*p* < 0.001) after SF@CP treatment. The level of NK1R in the conjunctiva of DED mice decreased by 44.00% (*p* < 0.0001) in the SF@CP@Gel group, 18.90% (*p* < 0.01) after CP treatment, and 25.92% (*p* < 0.5) after SF@CP treatment. Among them, the expression of SP and NK1R in both the cornea and conjunctiva after SF@CP@Gel treatment converged to the same level as that in the normal group.

### 2.7 SF@CP@Gel can Effectively Regulate the Th17/Treg Cell Balance

**Figure 7:**
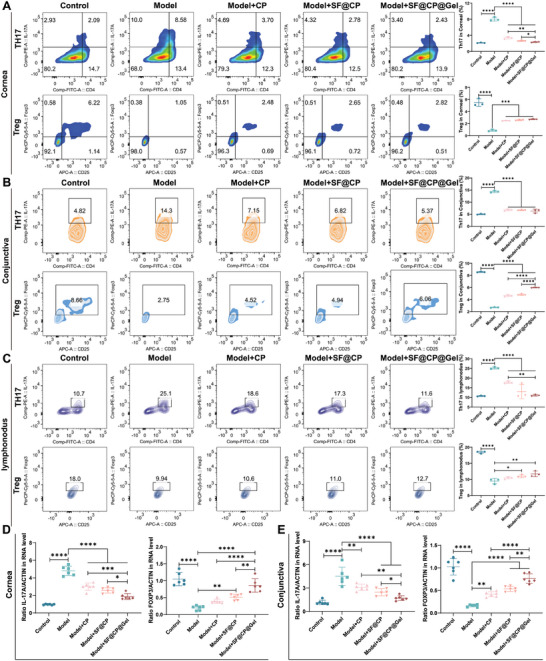
Flow cytometry analysis of Th17 and Treg cells and PCR analysis of IL‐17A and Foxp3 in different tissues. After induction of DED for 7 days, the mice received topical treatment with CP, SF@CP, or SF@CP@Gel for 14 days. The mice that received only 10 µl of PBS treatment composed the model group. A) The frequencies of IL‐17A[+]Th17 and Foxp3[+]Treg cells in corneas; DED mice treated with CP, SF@CP, SF@CP@Gel, or PBS; and untreated DED mice were evaluated on day 14 after DED induction using flow cytometry. B) The frequencies of IL‐17A[+]Th17 and Foxp3[+]Treg cells in the conjunctiva of DED mice treated with CP, SF@CP, SF@CP@Gel, or PBS, and untreated DED mice were evaluated on day 14 after DED induction using flow cytometry. C) The frequencies of IL‐17A[+]Th17 and Foxp3[+]Treg cells in the lymphatic nodes of DED mice treated with CP, SF@CP, SF@CP@Gel, or PBS, and untreated DED mice were evaluated on day 14 after DED induction using flow cytometry. D) RNA levels of IL‐17A and Foxp3 in the cornea of the control group, DED model group, CP treatment group, SF@CP treatment group, and SF@CP@Gel treatment group. E) RNA levels of IL‐17A and Foxp3 in the conjunctiva of the control group, DED model group, CP treatment group, SF@CP treatment group, and SF@CP@Gel treatment group (n = 14 mice per groups. One‐way ANOVA was used for the statistical analysis: *p < 0.05, **p < 0.01, ***p < 0.001, ****p < 0.0001; Data are presented as means ± SEM).

The percentages of Th17 and Treg cells in the cornea, conjunctiva, and lymph nodes were quantified using flow cytometry (**Figure** **7**). The frequency of CD4[+]IL‐17[+] T cells in the cornea, conjunctiva, and lymph nodes was significantly lower than that in the dry eye mouse model in all treatment groups (*p* < 0.0001). SF@CP@Gel had the greatest therapeutic effect on the DED model (in the cornea, a decrease of 73.56%; in the conjunctiva, a decrease of 64.01%; and in the lymph nodes, a decrease of 55.94%). In the cornea (Figure 7A), the frequency of CD4[+] IL‐17[+] T cells was significantly lower in the SF@CP@Gel treatment group than in both the CP treatment group (*p* < 0.001) and the SF@CP treatment group (*p* < 0.01). In the conjunctiva (Figure 7B), the percentage of CD4[+] IL‐17[+] T cells was significantly different among all three treatment groups. In the lymph nodes (Figure 7C), the decrease in Th17 cells in the SF@CP@Gel (*p* < 0.01) treatment group was significantly different from that in the CP treatment group (*p* < 0.01). Regrettably, the SF@CP@Gel treatment group did not have a significant advantage over the SF@CP treatment group. Changes in Treg cells are shown in Figure 7. After treatment with SF@CP@Gel, the frequencies of CD25[+] Foxp3[+] T cells in the cornea (3.11‐fold upregulation), conjunctiva (2.22‐fold upregulation), and lymph nodes (1.24‐fold upregulation) of DED mice were significantly increased. In the conjunctiva (Figure 7B), the upregulation of Tregs in the SF@CP@Gel treatment group was significantly different from that in both the CP group (*p* < 0.0001) and the SF@CP group (*p* < 0.0001). However, in the cornea and lymph nodes, the CD25[+] Foxp3[+] T‐cell frequency did not significantly differ among the three treatment groups yet still showed an increasing trend.

The expression of IL‐17A and Foxp3 are markers of Th17 and Treg cells identity, respectively; therefore, we examined the mRNA expression of the different transcription factors IL‐17A and Foxp3 in the cornea and conjunctiva by real‐time polymerase chain reaction (PCR) (Figure 7D,E). The expression of IL‐17A mRNA in the cornea in the SF@CP@Gel‐treated group decreased significantly compared with that in the DED model group (decreased by 65.34%, *p* < 0.0001), CP‐treated group (39.31% decrease, *p* < 0.001), and SF@CP‐treated group (46.21% decrease, *p* < 0.01). In the conjunctiva, the expression of IL‐17A mRNA in the SF@CP@Gel treatment group decreased by 55.04% (*p* < 0.001), 25.56% (*p* < 0.01), and 39.30% (*p* < 0.05) compared with that in the DED model group. The expression of Foxp3 mRNA in the cornea was significantly greater in the SF@CP@Gel treatment group than in the DED model group (6.81‐fold increase, *p* < 0.001), CP treatment group (2.48‐fold increase, *P* < 0.05), and SF@CP treatment group (3.05‐fold increase, *p* < 0.05). In the conjunctiva, the expression of Foxp3 mRNA in the SF@CP@Gel treatment group was 5.16 times greater than that in the DED group (*p* < 0.0001), 2.56 times greater than that in the CP treatment group (*p* < 0.01), and 3.38 times greater than that in the SF@CP treatment group (*p* < 0.05). In our study, the restoration of Treg cell frequency was not significant; however, the main manifestation was the elevation of the functional marker Foxp3.

## 3 Discussion

DED is a serious public health concern with a high prevalence worldwide. It affects vision‐related quality of life and causes an economic burden.^[^ ^31^ ^]^ In recent years, to effectively alleviate DED, a variety of anti‐inflammatory drugs have been developed with different targets. However, few drugs can simultaneously address inflammation and repair damaged ocular surfaces. In this study, we prepared a safe and stable ophthalmic gel (SF@CP@Gel) using an SF‐loaded NK1R antagonist and demonstrated its long‐term sustained release ability by a release experiment. After topical administration to DED mice for a sustained period of 14 days (twice a day), the NK1R antagonist CP effectively improved tear film stability and corneal epithelial damage in DED. The SF‐administered hydrogel enhanced the therapeutic effect of the CP drug and synergistically repaired the tear film and damaged corneal epithelium, consistent with the findings reported by Xie et al.^[^ ^32^ ^]^ Our study also demonstrated that incorporating SF nanoparticles into hydrogel systems can significantly enhance the sustained release properties of drugs. In addition, our results reaffirm that NK1R antagonists modulate Th17/Treg homeostasis to alleviate inflammation by blocking the SP/NK1R pathway. In summary, our study provides in vivo therapeutic evidence for targeting NK1R for the treatment of DED, with great potential for translation and application in future clinical treatment strategies.

In a variety of autoimmune diseases, Treg and Th17 cells can be transformed into each other by cytokines; however, the role of Th17/Treg homeostasis in ocular surface inflammation remains unclear. According to Yu et al.,^[^ ^17^ ^]^ NK1R antagonists alleviate the severity of DED by blocking APC‐mediated activation of Th17 cells. Some studies have further confirmed that NK1R antagonists also improved the severity of DED by restoring Treg function.^[^ ^15^ ^]^ These studies show that NK1R antagonists may ameliorate DED‐related inflammation by modulating the Th17/Treg balance. Nevertheless, the therapeutic effect of NK1R antagonists on DED and the transformation and application of ophthalmic preparations have yet to be comprehensively evaluated. In our in vivo study, SF@CP@Gel‐treated DED mice showed significant recovery of Treg cell function, as evidenced by the IL‐10, IL‐35, and TGF‐β1 levels. This finding was consistent with the trend of IL‐10 and TGF‐β1 changes in DLNs after in vivo treatment of DED mice with the NK1R antagonist Spantide I reported by Yukako et al.^[^ ^15^ ^]^ The quantitative analysis of Th17 and Treg cells in the cornea, conjunctiva, and lymph nodes suggested that SF@CP@Gel exhibited a greater ability to inhibit Th17 cells than to restore immunosuppressive Treg cells. The results of detecting the identity markers IL‐17A and Foxp3 in Th17 and Treg cells showed that the frequency of recovery of Treg cells was not significant but mainly manifested as an increase in the functional marker Foxp3. Therefore, we can infer that SF@CP@Gel ameliorated ocular surface inflammation mainly by suppressing the number and function of Th17 cells while restoring the function of Tregs. In addition, the expression levels of SP and NK1R in the cornea and conjunctiva further confirmed that SF@CP@Gel regulated ocular surface inflammation through the SP/NK1R signaling pathway. Our results verified the assumption that SF hydrogel‐loaded NK1R antagonists are long‐acting sustained‐release ocular formulations with anti‐inflammatory and restorative properties that ameliorate DED inflammation by modulating the Th17/Treg balance.

SF was approved by the Food and Drug Administration as a clinically acceptable biomaterial as early as 1993 and has been used in a variety of medical applications, including tissue regeneration, bone tissue scaffolds, and drug delivery.^[^ ^33^, ^34^, ^35^ ^]^ The use of SF‐encapsulated liposomes containing ibuprofen as ocular drugs demonstrated sustained drug release, permeation behavior, and good biocompatibility.^[^ ^36^ ^]^ Moreover, a previous study revealed that SF modulated lacrimal gland inflammation and ameliorated corneal and conjunctival abnormalities by increasing the number of conjunctival goblet cells in a DED mouse model.^[^ ^37^ ^]^ These results suggest that the SF drug delivery system on the ocular surface could not only provide sustained release and reduce toxicity but also regulate the intraocular immune microenvironment and repair corneal and conjunctival abnormalities, making it a promising ocular drug delivery system. In addition, the combined application of SF and hydrogel has excellent biocompatibility and excellent drug‐loading ability, and it can support the regeneration of corneal stromal tissue.^[^ ^24^, ^38^ ^]^ The primary structural sequence of SF contains many hydrophobic amino acid residues (GIy and Ala), which make it easy for SF molecules to undergo β‐folding and precipitate in aqueous solution.^[^ ^39^, ^40^, ^41^ ^]^ At the same time, some of the hydrophilic amino acid (Tyr and Ser) residues in the long side chain confer better water solubility to SF under specific conditions.^[^ ^41^, ^42^ ^]^ The block‐like structure of hydrophobic and hydrophilic chains in the amino acid sequence of SFs allows them to form micelle‐like structures in an aqueous solution.^[^ ^43^ ^]^ The CP drug can be encapsulated in the SF micellar cavity through noncovalent interactions (π‐π stacking and hydrogen bonding), resulting in SF@CP NPs.^[^ ^44^ ^]^ A large number of hydroxyl and amino groups on SF@CP NPs can cross‐link with Ca^2+^ gels via hydrogen bonding to form SF@CP@ gels.^[^ ^45^ ^]^ Our results demonstrated the superior therapeutic efficacy of the SF@CP@Gel treatment group compared with that of the CP treatment group and the SF@CP treatment group. Therefore, the CP drug modulates ocular surface inflammation, which was reinforced by the SF hydrogel.

Particle size and surface charge are critical parameters for the safe delivery of topical ocular formulations.^[^ ^21^ ^]^ We have shown that the size of the SF@CP drug‐loaded nanoparticles remained stable at 145 nm for 7 days, demonstrating their ability to circulate and achieve sustained release in vivo for a long period. Conventional ocular drop administration is characterized by a short residence time on the ocular surface, thus requiring multiple administrations. However, multiple administrations may increase drug side effects and decrease patient compliance. Therefore, ocular formulations providing long‐lasting release of the drug are necessary. In the present study, release experiments confirmed that SF@CP@Gel can fulfill the need for once‐a‐day administration. SF@CP@Gel continued to maintain the concentration of the drug for 25 h.

Daily exposure to intense UV rays and active metabolism may lead to a state of oxidative stress due to the imbalance between pro‐oxidants and antioxidants on the ocular surface, which is susceptible to damage by free radicals and ROS, triggering the activation of multiple inflammatory pathways.^[^ ^46^ ^]^ In recent years, there has been increasing evidence that oxidative stress plays an important role in the pathogenesis of different DEDs (dry and non‐dry DEDs as well as senile DEDs).^[^ ^47^ ^]^ The overexpression of ROS affects ocular surface health through multiple pathways, including lipid peroxidation of cell membranes in the lacrimal gland, promotion of inflammatory cell infiltration, and diminished antioxidant capacity of the corneal epithelium.^[^ ^48^, ^49^ ^]^ Therefore, antioxidant capacity is also an important factor for assessing the effectiveness of drug therapy. Our results clearly indicate that SF nanoparticles and gel materials have excellent safety and that CP and SF‐loaded CP gels can significantly improve antioxidant capacity and promote cell migration and proliferation. Our results confirmed the potential of this drug delivery system loaded with NK1R antagonists for clinical application in DED treatment and provided a valuable basis for future clinical translation. Future studies will focus on further elucidating the detailed mechanisms underlying the NK1R‐mediated signaling pathway and its role in DED pathogenesis. Specifically, investigating how NK1R modulation influences the balance between Th17 and Treg cells may provide deeper insights into the inflammatory processes of DED. Such mechanistic insights could pave the way for the development of more targeted therapeutic strategies.

## 4 Experimental Section

### Extraction and Purification of SF

The silk was boiled in 0.5% sodium carbonate solution for 30 min and then rinsed three times completely with deionized water. The above process was repeated twice. The three‐time degummed silk was drained, put into an oven at 80 °C for 3 h, and then dried to obtain the crude SF mixture. A mixture was prepared with a calcium chloride:ethanol:water molar mass ratio of 1:2:8. The SF mixture with a bath ratio of 1:20 was put into an 80 °C water bath and stirred for 2 h. The mixture was subsequently dissolved to obtain a mixture of SF and calcium chloride solution. After the mixture cooled to room temperature, it was transferred to a 14 000 Da dialysis bag and dialyzed with deionized water for 72 h, with water changes every 4 h. After the dialysis was completed, the SF solution was collected and centrifuged at 9000 °C for 15 min at 4 °C, and the insoluble materials were discarded. The liquid in the dialysis bag was dispensed into 50 mL centrifuge tubes and put into a freeze dryer (ALPHA 2–4 LD plus, Martin Christ, Germany). High‐purity SF was obtained by lyophilization.

### Preparation of SF NP

SF powder was mixed with MES solution (pH = 5, Sigma Aldrich, Shanghai, China) to form 1, 4, and 8 mg mL^−1^ solutions. An oil bath was heated at 120 °C for 1.5 h, the solution was cooled to room temperature, and corresponding ultra‐pure water was added. The mixture was then shaken at 37 °C and 150 r min^−1^ for 1 day, frozen at −80 °C for 8 h, thawed naturally, and dialyzed for 12 h to obtain a light‐blue transparent solution of SF NP. Among the three concentrations of SF NP, 1 mg mL^−1^ could not form nanoparticles well, 4 mg mL^−1^ formed nanoparticles with uniform size and good morphology, and 8 mg mL^−1^ formed nano‐aggregates with varying sizes and aggregation. Therefore, 4 mg mL^−1^ was finally chosen for the preparation of SF NP (Figure S1, Supporting Information).

### Preparation of SF@CP NP Hydrogels

SF powder was combined with MES solution (pH = 5, Sigma Aldrich, Shanghai, China) to form a solution. An oil bath was heated at 120 °C for 1.5 h, the SF solution was cooled to room temperature, and CP solution was added in the ratio of 8:1, 4:1, and 2:1. The mixture was then shaken at 37 °C and 150 r min^−1^ for 1 days, frozen at −80 °C for 8 h, thawed naturally, and dialyzed for 12 h to obtain three concentrations of light‐blue transparent solution of SF@CP NPs. Among the three concentrations of SF@CP NPs, the ratio of 8:1 was small, could not form nanoparticles well, and was severely adhered, and the ratio of 2:1 particles was large and severely adhered (Figure S2, Supporting Information). Therefore, the ratio of 4:1 was chosen for the preparation of SF@CP NP hydrogels (synthesized by 4 mg mL^−1^ SF solution with 1 mg mL^−1^ CP solution).

A 1% sodium alginate aqueous solution (solution A) and a 0.5% calcium gluconate solution (solution B) were prepared. The AB solution was mixed ultrasonically at a volume ratio of 1:0.36 to form ionic gels. The SF@CP NP solution was blended with 2% SA solution at a volume ratio of 1:1 by ultrasonication and then blended with AB solution at a volume ratio of 1:0.36 by ultrasonication to obtain a drug‐carrying SF gel (SF@CP NP hydrogel). All the samples were freeze‐dried at −50 °C for 48 h.

### Physicochemical Properties of Drug‐Loaded SF Nanoparticles

The particle size and morphology of SF@CP were separately measured and characterized by DLS (Zetasizer Nano ZS90, Malvern, UK), TEM (Talos L120C, Thermo Fisher, Germany), and SEM (Sigma500, Zeiss, Germany). The size stability of SF@CP was tested via UV spectral analysis. To confirm the successful loading of the CP drug on SF, the zeta potential was evaluated (Zetasizer Nano ZS90, Malvern, UK), and UV spectroscopy (UV‐2600i, SHIMADZU, Japan) was used. Finally, the drug loading and encapsulation rates were measured using UV spectrophotometry. The CP drug was first prepared as a 1 mg mL^−1^ master batch and then diluted by the corresponding multiples. The absorbance at the maximum absorption wavelength of 275 nm was measured by a UV‐2600i UV–visible spectrophotometer and plotted as the standard working curve of the CP concentration. SF powder was combined with MES solution (Sigma Aldrich, Shanghai, China) (pH = 5) to form a 4 mg mL^−1^ solution. An oil bath was heated at 120 °C for 1.5 h, the solution was cooled to room temperature, and 1 mg mL^−1^ CP solution was added. The solution was then shaken at 37 °C and 150 r min^−1^ for 1 days, frozen at −80 °C for 8 h, thawed naturally, and dialyzed for 12 h to obtain a light blue transparent SF@CP NP solution. The dialysate was collected, and the volume was calculated. The dialysis solution was diluted appropriately, and the absorbance of the dialysis solution was tested by UV‐2600i, which was substituted into the standard curve to calculate the mass of the drug in the dialysis filtrate. Then, the mass of the loaded drug was calculated according to the difference method. The encapsulation capacity and loading efficiency were calculated according to the following equations: Encapsulation capacity = mass of encapsulated drug/total drug dosage; Loading efficiency = mass of drug encapsulated/mass of drug encapsulated + mass of carrier.

### Physicochemical Properties of the Drug‐Loaded SF Gel

The microstructure and molecular structure of the gel were determined by SEM and NMR spectroscopy (AVANCE III 600 MHz, Bruker, Germany). The properties of hydrogels are influenced by their rheological characteristics, and the deformation and flow of the material may undergo time‐dependent changes under different external conditions, such as stress, strain, or temperature. A 15‐mL sample of hydrogel was used to characterize the rheological properties of the hydrogel using a micro infrared rheometer (MARS60, Thermo Fisher, Germany), and oscillatory rate sweep tests were performed at 25 °C ± 0.2 °C with γ = 0%–1000% and f = 1 Hz. The temperature (25 °C), strain (γ = 1%), and frequency (1 Hz) were fixed to obtain viscosity‐time relationship curves. Solubilization without dissolution is an important property of gel materials; its solubilization properties were measured using the tea‐bag method. In addition, we used UV spectrophotometry to determine drug release from this drug delivery system. Two milliliters of the carrier system were sealed in a dialysis bag and shaken continuously at 37 °C. Every 2 h, 2 mL of the solution was removed and centrifuged, while 2 mL of the corresponding PBS was added to the centrifugation product. The absorbance at 275 nm in the supernatant was determined by UV spectroscopy by calculating the release at different times based on the standard curve. The cumulative method was used to calculate the release at different times, hence the release rate.

### In Vitro Cytotoxicity Assays

The biocompatibility of the materials was assessed using the CCK‐8 assay (Selleck, B34302, China). HCECs were purchased from Procell (CL‐0743, Wuhan, China). HCECs were analyzed using DMEM/F12 (Gibco, 12 634 010, USA), 15% fetal bovine serum (FBS, Gibco, 10099141C, New York, USA), 5 µg mL^−1^ insulin (Procell, PB180432, Wuhan, China), 10 ng mL^−1^ human epidermal growth factor (PCK001, China), and 1% penicillin–streptomycin (PB180120, China) and were cultured at 37 °C in a 5% CO2 incubator. HCECs were inoculated in 96‐well plates at a density of 1 × 104 cells per well and incubated for 24 h. Furthermore, 1 mg mL^−1^ SF NPs, SF@CP@Gel hydrogels, and different concentrations of SF@CP@Gel extracts prepared in advance were added and incubated for 24 h. Subsequently, CCK‐8 reagent was added, and the cells were incubated for 0, 24, 48, 72, or 96 h. We assessed cell survival by measuring the OD at 450 nm. The experiment was repeated three times independently.

### Cell Antioxidant and Migration Ability Tests

Oxidative stress was induced in well‐grown HCECs using LPS. The content of ROS was measured by DCFH‐DA to assess the degree of cellular oxidative damage. HCECs were inoculated in 48‐well plates at a density of 3.5 × 10^4^ cells per well and treated with 1 mg mL^−1^ LPS for 24 h. The drugs were then removed from the cell cultures and treated with CP or the SF@CP@Gel hydrogel for 24 h. The HCECs were then incubated with 20 µM DCFH‐DA solution (Thermo Fisher Scientific, Massachusetts, USA) for 30 min at 37 °C, and the cells were analyzed using a fluorescence microscope (Infinite M200, Inc., Tecan, Switzerland) for green fluorescence imaging. The fluorescence intensity was measured by flow cytometry (Becton Dickinson). Untreated cells were used as controls. The results are expressed as the MFI of DCF.

HCECs were inoculated in 6‐well plates at a density of 5 × 10^4^ cells per well and grown to 90% confluence. After that, a sterile 200 µl plastic pipette tip was used to make scratches across the cell surface to create artificial scratches. Dissociated cells were removed by washing with PBS (0.01 M PBS, pH = 7.4) (Sigma Aldrich, Shanghai, China). CP and SF@CP@Gel hydrogels were added for coincubation, and images of the same scratch area were taken at 0, 6, 18, and 24 h to determine scratch closure. The scratch area was measured using ImageJ. The formula for calculating the cell migration rate was (1 – scratch area at the specified time/initial area of the scratch) × 100%.

### Construction and Evaluation of the Dry Eye Animal Model

Seventy female C57BL/6 mice (weighing 18 ± 2 g, aged 6–8 weeks) were purchased from Beijing Viton Lihua Laboratory Animal Technology Co. All animal experiments were conducted by the Association for Research in Vision and Ophthalmology Statement on the Use of Animals in Ophthalmic and Vision Research. The ethical approval for the human study was obtained from the Medical Ethics Committee of the Sichuan Academy of Medical Sciences, Sichuan Provincial People's Hospital (approval number: 2023–210). The animals were kept in an environment with a temperature of 20–25 °C and a relative humidity of 30%–70%. The dry eye animal model was constructed by applying 0.3% benzalkonium chloride solution to both eyes at regular intervals every day after the animals were subjected to hot air blowing under a hot air blower for 40 min, and this process lasted for 7 days.

### Evaluation of Treatment Effects

After initial screening to exclude existing ocular surface diseases, the mice were randomly divided into five groups: a control group, a model group, and three treatment groups (CP treatment group, SF@CP treatment group, and SF@CP@Gel treatment group). There were 14 mice in each group. Mice that did not receive any treatment composed the normal control group, and those that successfully established a dry eye model and received only 10 µl of PBS treatment composed the model group. The drug was administered topically to both eyes twice a day (morning and evening) at a volume of 10 µl once every 14 days. Clinical assessments were performed simultaneously (3 pm) on days 0, 3, 7, and 14 of treatment.

A 1‐mm phenol red cotton thread was placed in the conjunctival fornix at the lateral canthus of the lower lid of the mice for 30 s. The length of the reddening of the phenol red cotton thread was measured with a Vernier caliper to assess the amount of tear secretion, and the degree of corneal epithelial damage was assessed by CFS, in which 1 µl of 2.5% fluorescein was applied to the conjunctival sac of the mice. The scoring method was based on the NEI criteria; the cornea was divided into five zones, and each zone was scored on a scale of 0–3.

The IL‐10, IL‐35, and TGF‐β1 levels in conjunctival tissues were measured using ELISA. Conjunctival tissues were collected from each group and added to lysis buffer (Thermo Fisher Scientific, Massachusetts, USA, 36 978) containing a protease inhibitor (Thermo Fisher Scientific, Massachusetts, USA, 78 510). The tissues were fully lysed for 30 min and centrifuged at 16 000 × g for 30 min at 4 °C. The supernatant was collected according to the IL‐10 (CUSABIO, China, CSB‐E04594m), IL‐35 (CUSABIO, China, CSB‐E13145m), and TGF‐β1 (CUSABIO, China, CSB‐E04726m) levels and quantified according to the manufacturer's ELISA kit protocol.

### Histological Analysis and Apoptosis Assessment

To further assess the therapeutic effect of SF@CP@Gel, the mice were sacrificed at the end of the experiment. The eyeballs, corneas, and conjunctiva were collected for histological examination. The specimens were fixed in 4% paraformaldehyde (Beyotime, China). Paraffin‐embedded sections of dehydrated specimens were prepared. After the paraffin sections were dewaxed, hydrated, and stained with hematoxylin and eosin to visualize the nuclei and cytoplasm, respectively, the sections were dehydrated, sealed, and then photographed under a light microscope to observe morphological changes. A Masson's trichrome stain kit (Baso, Zhuhai, China) was used. Masson staining was performed according to the manufacturer's instructions. Apoptosis was detected using an in situ cell death detection kit (Roche, Basel, Switzerland). After the tissues were treated with proteinase K, 50 µl of TUNEL reaction mix (50 µl of TdT + 450 µl of fluorescein‐labeled dUTP solution) was added, and the samples were incubated for 1 h at 37 °C in a dark wet box. For the negative controls, 50 µl of a fluorescein‐labeled dUTP solution was added. Converter‐peroxidase horseradish peroxidase labeling solution was added; a DAB substrate kit was used to develop the color, and hematoxylin was used to restain, clear, and seal the plate. Finally, DAPI was used to visualize the nucleus. The apoptotic cells were counted under a fluorescence microscope (Infinite M200, Tecan, Deacon, Switzerland) (excitation wavelength 450–500 nm and detection wavelength 515–565 nm). The number of DAPI‐ and TUNEL‐positive puncta was calculated as the total number of cells by ImageJ software, and the number of TUNEL‐positive puncta was used as the cutoff point. Finally, TUNEL/DAPI × 100% was used to calculate the percentage of apoptotic cells. The stained sections were all observed and imaged using a light microscope (Olympus, Tokyo, Japan).

### Th17 and Treg Percentage Assay by Flow Cytometry

The percentages of Th17 and Treg cells in the cornea, conjunctiva, and lymph nodes were quantified using flow cytometry. Corneal and conjunctival tissues were carefully dissected and cut into ≈1 mm^3^ pieces. These tissues were enzymatically digested using a solution of collagenase IV (1 mg mL^−1^) and DNase I (0.1 mg mL^−1^) in RPMI‐1640 medium for 45 min at 37 °C in a shaking water bath to ensure optimal dissociation. Lymph nodes were gently mechanically dissociated using a tissue grinder and subjected to the same enzymatic treatment. After digestion, the cell suspensions were passed through a 70‐um nylon cell strainer to remove undigested tissue fragments. The filtered cells were then centrifuged at 300 g for 5 min at 4 °C, washed with PBS, and resuspended in PBS containing 2% FBS. Red blood cell lysis was performed when necessary using an ammonium–chloride–potassium lysis buffer for 3 min at room temperature. The resulting single‐cell suspensions were used for staining and flow cytometry analysis. A total of 1 × 10^7^ corneal, conjunctival, and lymph node cells were centrifuged in a 1.5 ml centrifuge tube at 3000 r min^−1^ for 5 min, and the supernatant was discarded. A single‐cell suspension was prepared by adding 100 ul of fluorescent‐activated cell sorting (FACS) buffer to suspend the cells. The gating strategy was carefully designed to ensure the accurate identification of specific cell populations. Initially, lymphocytes were identified based on forward scatter (FSC) and side scatter (SSC) characteristics to isolate cells of appropriate size and granularity. Next, doublets were excluded by plotting FSC‐A against FSC‐H to ensure that only single cells were analyzed. To further refine the population, live cells were gated by excluding those positive for the Live/Dead viability dye. From the live cell population, CD4^+^ T cells were gated, followed by additional gating to identify CD25^+^ Foxp3^+^ Treg cells and IL‐17^+^ Th17 cells. These cell populations were then analyzed for their respective marker expression to ensure accurate and reliable results. An anti‐mouse IgG Fc antibody (0.05 µg mL^−1^ Abcam, ab197780) was added to the water bath for 3 min. BD Pharmingen FITC Rat Anti‐Mouse CD4 (0.5 mg mL^−1^, Bioscience, 553 729), PE anti‐mouse IL‐17A antibody (0.2 mg mL^−1^, Biolegend, 506 903), APC anti‐mouse CD25 recombinant antibody (0.2 mg mL^−1^, Biolegend, 162 105), BD Pharmingen PerCP‐Cy 5.5, and rat anti‐mouse Foxp3 (0.2 mg mL^−1^, Bioscience, 563 902) were also added to the water bath for 30 min. Then, 350 µl of FACS buffer was added, the mixture was gently mixed, and the mixture was centrifuged at 3000 r min^−1^ for 5 min. The supernatant was discarded. The process was repeated twice to remove the free fluorescent antibody. Then, 100 ul of FACS buffer was added to the obtained cell precipitate, and the cells were gently mixed to suspend the cells. The cell suspension was transferred to a FACS‐specific tube for detection and analysis on a flow cytometer (Beckman Coulter, Inc., Pasadena, CA, USA).

### Assessment of the mRNA Levels of IL‐17 and FoxP3 by Quantitative Real‐Time PCR

The mRNA levels of IL‐17 and FoxP3 in corneal and conjunctival tissues were assessed by qRT‐PCR. Tissues were stored frozen at −80 °C in Qiazol's solution. Total RNA was extracted using the miScript SYBR Green PCR Kit (DP501) and reverse transcribed using the miScript II RT Kit. Real‐time PCR was performed using TaqMan Universal PCR Master Mix with predesigned primers (for IL‐17A and FoxP3). The samples were analyzed using a real‐time fluorescent quantitative PCR system (LightCycler 96). Relative expression levels were normalized to those of. The relative mRNA expression was calculated using the 2^−ΔΔCT^ method. The primers used are shown in Table S1.

### Fluorescence Images and Quantitative Analysis of ROS Levels in Ocular Tissues

After harvesting fresh eyeballs, the gauze was dried of moisture and fixed and dehydrated by rapid freezing in liquid nitrogen for 15 s. The specimens were then embedded using an OCT embedding medium (Thermo, NEG‐50) and sliced with a microtome to a thickness of 8–10 um, placed on glass slides. The slices were dried at room temperature for 30–60 min, followed by fixation in 4% paraformaldehyde for 2–3 min and washes in PBS (pH 7.4) three times, each for 5 min. Dihydroethidium staining solution (1:200, Biyuntian, S0063) was added to the tissue slices and incubated at 37 °C for 30 min. After incubation, the slides were placed in PBS (pH 7.4) and washed on a decolorization shaker three times, each for 5 min. After gently shaking off excess liquid, DAPI staining solution (Wknow, WK00020) was added within the circles and incubated at room temperature in the dark for 5 min. The slides were again washed in PBS (pH 7.4) on the decolorization shaker three times, each for 5 min. The slices were gently shaken dry and then mounted with an anti‐fluorescence quenching mounting medium (Wknow, WK11003). Finally, the slices were placed under a digital slide scanner (3DHISTECH, PANNORAMIC SCAN 150) for image acquisition.

### Assessment of SP and NK1R Content by Immunohistochemistry

Specimen tissue sections were deparaffinized, hydrated, and immersed in 3% H2O2 methanol for 10 min to remove endogenous catalase. Serum closure was performed after high‐pressure antigen retrieval. Then, 4 µg mL^−1^ of anti‐NK1R antibody (Abcam, ab219600) or anti‐SP antibody (Abcam, ab14184) was added at a dilution of 1:1000 and stored overnight. After washing with PBS, biotinylated goat anti‐rabbit IgG (H+L) (Abcam, ab64256) or biotinylated goat anti‐mouse IgG (H+L) (Abcam, ab64255) was added at a dilution of 1:200 and incubated for 0.5 h at room temperature. After the slices were incubated with DAPI for 2 min, they were blocked. Sections were observed and photographed under a fluorescence microscope (Infinite M200, TECAN, Deacon, Switzerland) and quantitatively analyzed to obtain the IOD using ImageJ software (National Institutes of Health, Bethesda, MD).

### Statistical Analysis

The data were analyzed using SPSS v.17.0 statistical software (IBM, USA). Data pre‐processing involved evaluating outliers using the Grubb’ test. Data are presented as means ± SEM, and all statistical analyses were based on a minimum of three samples per group. Statistical methods included One‐way ANOVA and Two‐way ANOVA. Statistical significance was defined as a *p*‐value < 0.05.

## Conflict of Interest

The authors declare no conflict of interest.

## Supporting information



Supporting Information

## Data Availability

The data that support the findings of this study are available in the supplementary material of this article.
