# Supplementary material for: A Silk Fibroin Nanoparticle Hydrogel Loaded With NK1R Antagonist Has Synergistic Anti‐Inflammatory and Reparative Effects on Dry Eye Disease
Source: Adv Sci (Weinh). 2025 Feb 22;12(15):2404835. doi: 10.1002/advs.202404835 (PMC12005769; doi:10.1002/advs.202404835)
Supplement: Supplementary file 1 — Supporting Information [file ADVS-12-2404835-s001.docx]

Supporting Information

A Silk Fibroin Nanoparticle Hydrogel Loaded with NK1R Antagonist Has Synergistic Anti-Inflammatory and Reparative Effects on Dry Eye Disease

*Bo Gong, Yi Liu, Huan Li, Xueming Ju, Dongfeng Li, Yuhao Zou, Xiaoxin Guo, Kai Dong, Jialing Xiao, Weijia Wu, Renjie Chai *, Ruifan Zhang*, Man Yu **

Table S1

| Primer name | Sequence (5' to 3') | Amplicon size |
| --- | --- | --- |
| IL-17A-F | TACCTCAACCGTTCCACGTC | 119 |
| IL-17A-R | TTTCCCTCCGCATTGACACA | 119 |
| FOXP3-F | GGGGAAGCCATGGCAATAGT | 199 |
| FOXP3-R | GCGGGGTGGTTTCTGAAGTA | 199 |
| β-actin-F | ACCCTAAGGCCAACCGTGAAA | 193 |
| β-actin-R | ATGGCGTGAGGGAGAGCATA | 193 |


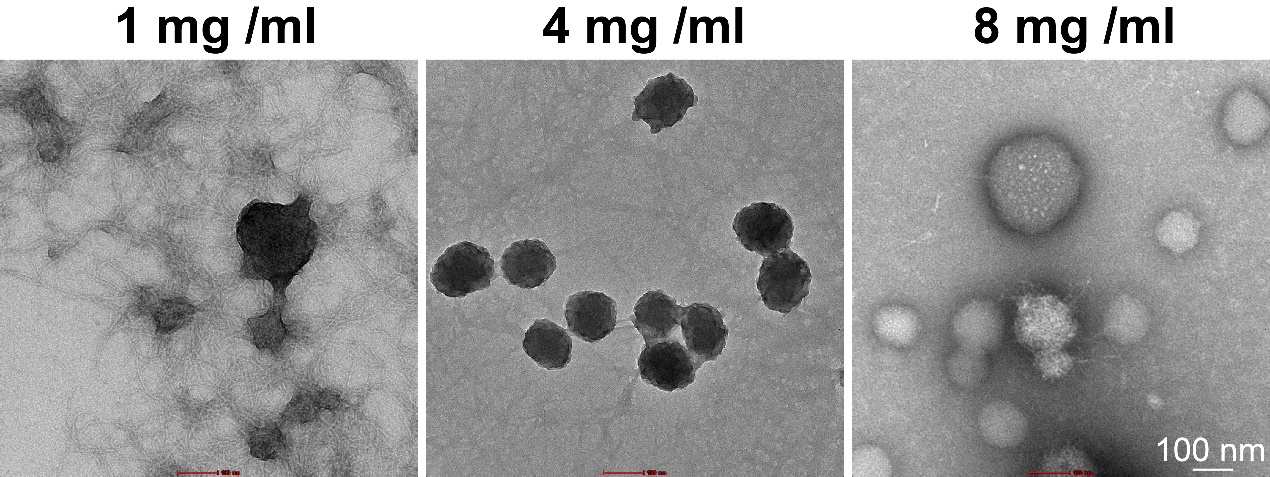


**Figure S1**. SF NP particles in different concentrations.


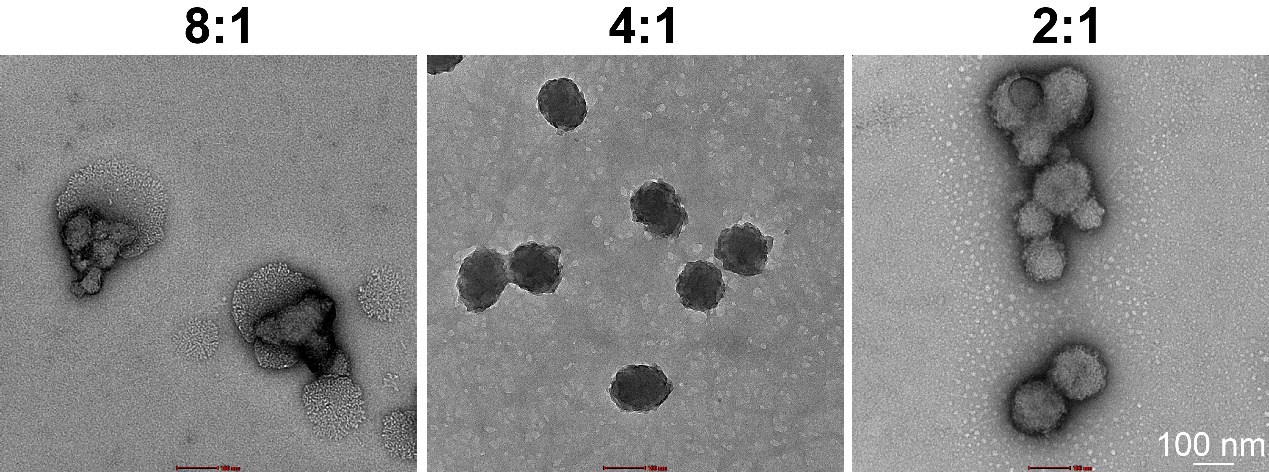


**Figure S2.** The SF@CP particles were synthesized in 8:1, 4:1 and 2:1 ratio, respectively.


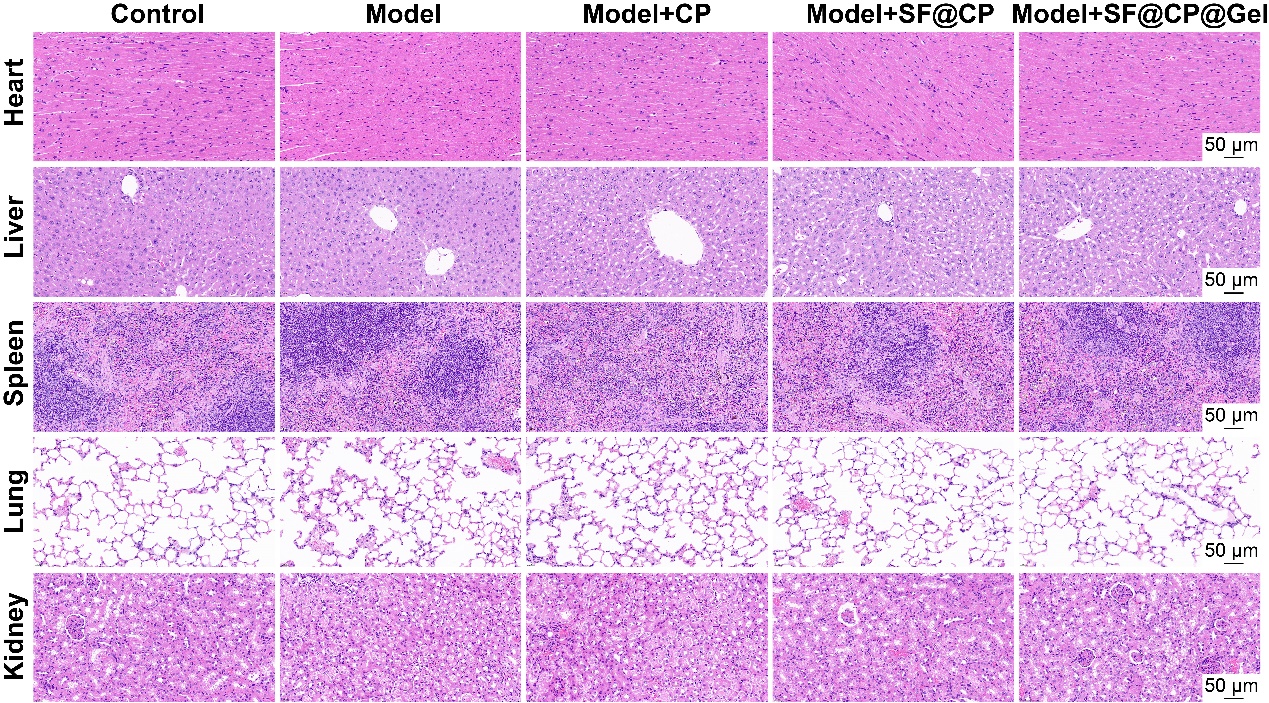


**Figure S3.** Hematoxylin-eosin staining of the heart, liver, spleen, lung and kidney from the control group, DED model group, CP treatment group, SF@CP treatment group, and SF@CP@Gel treatment group.


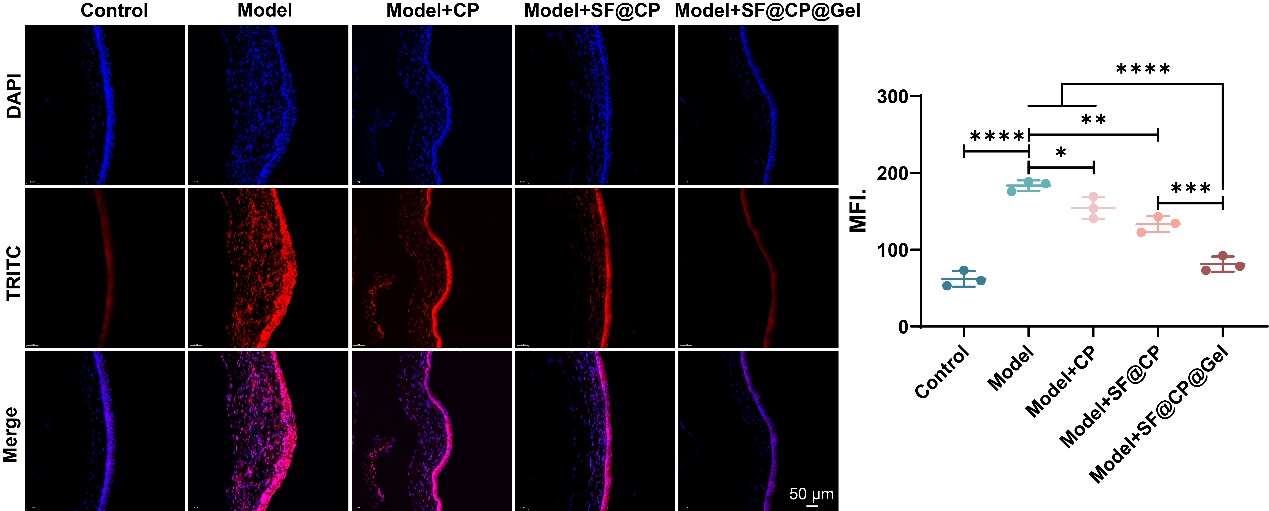


**Figure S4**. Fluorescence diagram and quantitative analysis of ROS level in eyeball tissue. The nucleus of DAPI staining was blue, and the positive expression of ROS was red labeled with the corresponding fluorescein. n=3.
